# Oculo-vestibular signs in experimentally induced migraine attacks: an exploratory analysis

**DOI:** 10.1007/s10072-022-06312-4

**Published:** 2022-08-12

**Authors:** Michele Corrado, Chiara Demartini, Rosaria Greco, Annamaria Zanaboni, Federico Bighiani, Gloria Vaghi, Valentina Grillo, Grazia Sances, Marta Allena, Cristina Tassorelli, Roberto De Icco

**Affiliations:** 1 Department of Brain and Behavioral Sciences, University of Pavia, Pavia, Italy; 2 Headache Science and Neurorehabilitation Center, IRCCS Mondino Foundation, Pavia, Italy

**Keywords:** Human migraine models, Pain, Nitroglycerin, Nystagmus, Head Impulse Test, Vertigo

## Abstract

Vestibular symptoms accompanying headache are quite common in migraine patients. Based on the association of vertigo with migraine, vestibular migraine was included in the appendix of the 3rd edition of the International Classification of Headache Disorders as a possible migraine subtype worthy of further investigation. In this post hoc, exploratory analysis, we investigated the occurrence of oculo-vestibular signs (OVSs) during experimentally induced migraine attacks in 24 episodic migraine patients and 19 healthy controls exposed to sublingual nitroglycerin (NTG - 0.9 mg). A comprehensive clinical examination was performed at baseline, at the onset of the migraine-like attack, and immediately before hospital discharge (180 minutes after NTG administration). Three of the 13 migraine patients who developed a spontaneous-like migraine attack during the hospital observation period (23.1%) also developed OVSs during the induction test. Noteworthy, none of the patients with a negative induction test developed OVSs and no OVSs were reported in healthy subjects at any time point. The exploratory nature of our study does not allow to draw definite conclusions on the possible implications of a vestibular dysfunction in migraine pathophysiology. Our results however suggest that NTG administration may lend itself to investigate vestibular dysfunction in migraine, at least in a subset of patients. The present findings represent a starting point for designing future ad hoc and well-powered studies.

## Introduction

Migraine attacks may be associated to vestibular symptoms and signs. Vestibular symptoms occurring during a migraine attack are considered by some authors one of the most common vestibular syndromes [1]. The International Classification of Headache Disorders (ICHD-3) has proposed diagnostic criteria for vestibular migraine (VM) that require the presence of vestibular symptoms of moderate or severe intensity, lasting between 5 minutes and 72 hours, in patients with a history of migraine; for the the diagnosis, it is mandatory that at least half of episodes are associated with one or more of the following: headache with migraine features, photophobia and phonophobia, or visual aura [2].

Neurological examination performed during attacks of VM has detected transient signs of oculo-vestibular dysfunction, which usually disappear outside of the VM attack [3]. These oculo-vestibular signs (OVSs) include spontaneous or positional central nystagmus, head shaking–induced nystagmus, and peripheral types of nystagmus with vestibular deficits [3–5]. Vestibular signs have been described also interictally in patients with VM and in subjects with migraine without vestibular symptoms [6, 7]. Thus, it is possible that vestibular dysfunction is part of migraine pathophysiology in general.

Here we present a post hoc, exploratory analysis of a previously published double-blind, parallel-group controlled study where we investigated the occurrence of OVSs during experimentally induced migraine attacks in migraine patients without VM exposed to sublingual nitroglycerin (NTG). The aim of this exploratory report is to provide preliminary data for future well-powered confirmatory studies.

## Methods

The full description of the study population and experimental design is reported in the main study [8]. Briefly, our sample comprised 24 patients with a diagnosis of episodic migraine (MIG group) and a group of 19 healthy controls (HC group). None of the subjects fulfilled ICHD-3 appendix criteria for VM.

All subjects received sublingual NTG administration (0.9 mg) and were kept under observation at the IRCCS Mondino Foundation (Pavia, Italy) for 180 minutes.

The NTG-based migraine induction test was rated “positive” (MIG + group) in all subjects who developed a migraine-like attack that met the criteria for experimentally induced migraine headache in the 12-hour period after NTG administration. The study was approved by the local Ethics Committee (14/int/2016).

### Oculo-vestibular examination

This consisted in a thorough clinical evaluations of ocular movements [9], and the following parameters were included as OVSs in the database: evaluation of Head Impulse Test (HIT), nystagmus with and without visual fixation, and test of Skew with cover test (according to the HINTS examination, a validated and sensitive tool for detecting central vestibular dysfunction [10, 11]; evaluation of smooth pursuit, vergence, saccadic ocular movements, and Head Shaking Test (HST). Modification or appearance of nystagmus in the position of Rose (supine with head hanging down) were also assessed [12].

The examination was performed by an expert neurologist (RDI) on all subjects at baseline and before hospital discharge (T-180 post-NTG administration). In the MIG + subjects with a migraine-like attack occurring within the 180-minute observation period, the oculo-vestibular examination was repeated at migraine onset (T-MIG +).

## Results

Sixteen migraine patients developed a migraine-like headache; in 13 of them, the onset occurred during the 180-minute observation period.

At baseline, one subject showed a down-beating nystagmus, not modified by visual fixation or positional changes. In this patient (ID = 15), nystagmus persisted unchanged at migraine-like headache onset (T-MIG +) and was still present at the moment of hospital discharge (T-180).

Three migraine patients (12.5%) developed new-onset OVSs during the induction test at T-MIG + . The documented OVSs were still present at T-180 in all three patients (Table 1). Of these, two subjects (ID = 2 and ID = 17) had a down-beating nystagmus, which was not modified by visual fixation or Rose position. The third subject (ID = 21) showed a positive HIT with overt saccades; in this subject, a left-beating nystagmus evoked by the HST was also observed.

**Table 1:** Vestibular signs in migraine patients during the nitroglycerin induction test

ID	Migraine-like headache	Latency from NTG (min)	Baseline										Migraine-like onset										180 minutes from NTG									
			Ny-Fix	Ny-No Fix	Ny–Fix-Rose	Ny–No Fix-Rose	Vergence	SP	Saccades	HST	HIT	TS	Ny-Fix	Ny–No Fix	Ny–Fix-Rose	Ny–No Fix-Rose	Vergence	SP	Saccades	HST	HIT	TS	Ny-Fix	Ny – No Fix	Ny – Fix - Rose	Ny – No Fix - Rose	Vergence	SP	Saccades	HST	HIT	TS
1	N		-	-	-	-	-	-	-	-	-	-											-	-	-	-	-	-	-	-	-	-
2	Y	25	-	-	-	-	-	-	-	-	-	-	-	DB	-	DB	-	-	-	-	-	-	-	DB	-	DB	-	-	-	-	-	-
3	Y	25	-	-	-	-	-	-	-	-	-	-	-	-	-	-	-	-	-	-	-	-	-	-	-	-	-	-	-	-	-	-
4	Y	30	-	-	-	-	-	-	-	-	-	-	-	-	-	-	-	-	-	-	-	-	-	-	-	-	-	-	-	-	-	-
5	N		-	-	-	-	-	-	-	-	-	-											-	-	-	-	-	-	-	-	-	-
6	N		-	-	-	-	-	-	-	-	-	-											-	-	-	-	-	-	-	-	-	-
7	N		-	-	-	-	-	-	-	-	-	-											-	-	-	-	-	-	-	-	-	-
8	Y	120	-	-	-	-	-	-	-	-	-	-	-	-	-	-	-	-	-	-	-	-	-	-	-	-	-	-	-	-	-	-
9	Y	180	-	-	-	-	-	-	-	-	-	-	-	-	-	-	-	-	-	-	-	-	-	-	-	-	-	-	-	-	-	-
10	N		-	-	-	-	-	-	-	-	-	-											-	-	-	-	-	-	-	-	-	-
11	N		-	-	-	-	-	-	-	-	-	-											-	-	-	-	-	-	-	-	-	-
12	N		-	-	-	-	-	-	-	-	-	-											-	-	-	-	-	-	-	-	-	-
13	N		-	-	-	-	-	-	-	-	-	-											-	-	-	-	-	-	-	-	-	-
14	Y	360	-	-	-	-	-	-	-	-	-	-											-	-	-	-	-	-	-	-	-	-
15	Y	30	-	DB	-	DB	-	-	-	-	-	-	-	DB	-	DB	-	-	-	-	-	-	-	DB	-	DB	-	-	-	-	-	-
16	Y	30	-	-	-	-	-	-	-	-	-	-	-	-	-	-	-	-	-	-	-	-	-	-	-	-	-	-	-	-	-	-
17	Y	40	-	-	-	-	-	-	-	-	-	-	-	DB	-	DB	-	-	-	-	-	-	-	DB	-	DB	-	-	-	-	-	-
18	Y	75	-	-	-	-	-	-	-	-	-	-	-	-	-	-	-	-	-	-	-	-	-	-	-	-	-	-	-	-	-	-
19	Y	240	-	-	-	-	-	-	-	-	-	-											-	-	-	-	-	-	-	-	-	-
20	Y	60	-	-	-	-	-	-	-	-	-	-	-	-	-	-	-	-	-	-	-	-	-	-	-	-	-	-	-	-	-	-
21	Y	25	-	-	-	-	-	-	-	-	-	-	-	-	-	-	-	-	-	←	Bil	-	-	-	-	-	-	-	-	←	Bil	-
22	Y	30	-	-	-	-	-	-	-	-	-	-	-	-	-	-	-	-	-	-	-	-	-	-	-	-	-	-	-	-	-	-
23	Y	55	-	-	-	-	-	-	-	-	-	-	-	-	-	-	-	-	-	-	-	-	-	-	-	-	-	-	-	-	-	-
24	Y	240	-	-	-	-	-	-	-	-	-	-											-	-	-	-	-	-	-	-	-	-

*Ny-Fix*, nystagmus with fixation; *Ny-No Fix*, nystagmus without fixation; *Ny-Fix-Rose*, nystagmus with fixation evaluated in Rose position; *Ny-No Fix-Rose*, nystagmus without fixation evaluated in Rose position; *SP*, smooth pursuit; *HST*, Head Shaking Test; *HIT*, Head Impulse Test; *TS*, test of Skew and cover test; *DB*, down-beating nystagmus; *Bil*, bilateral vestibular hypofunction; ← , left-beating nystagmus

None of the patients with a negative induction test (MIG- group) developed OVSs throughout the study (Table 1). Of note, none of the 4 subjects with OVSs reported subjective oculo-vestibular symptoms during the observation period.

Finally, we did not find any OVSs in the HC group across all time points.

## Discussion

VM has been proposed as one of the most common recurrent vestibular syndromes, but its pathophysiology is poorly known. Abnormal oculo-vestibular function has been detected in a large portion of patients with VM. It must be noted that OVSs are not specific for VM, as they can also be present in patients with non-vestibular migraine phenotypes [7, 13].

Human migraine models based on the administration of migraine-provoking agents represent validated tools to assess features of the acute migraine phase as they allow to reliably induce spontaneous migraine attacks in a consistent percentage of migraine patients [8, 14].

In this exploratory post hoc analysis, we described the occurrence of OVSs during experimentally NTG-induced migraine attacks. Our study was conducted in a small population and OVSs were observed in a minority of migraine subjects; however, we believe that our findings may be important because: i) we evaluated a population of migraine subjects without an oculo-vestibular phenotype; ii) OVSs were observed exclusively in the migraine subjects who developed a migraine attack after the NTG challenge; iii) none of the subjects with OVSs reported subjective occurrence of oculo-vestibular symptoms; and iv) the OVSs described in this study are in line with previous literature [3, 6].

These observations suggest that an abnormal oculo-vestibular function is an underlying trait of at least a subset of migraine patients without a VM phenotype, and point to a role of the oculo-vestibular system in migraine, probably not limited to VM.

Although exploratory, our findings may be useful for providing the basis to plan future well-powered studies. Testing vestibular function in VM and/or non-VM under controlled conditions may indeed contribute to widen our knowledge of migraine pathophysiology and to better characterize migraine phenotypes.

Our study suffers several limitations, which are anyway expected in a post hoc, exploratory study design. First, the vestibular testing was performed by a single unblinded neurologist, and we are aware that a double-blind design on a large population is needed to confirm the validity of our findings. Moreover, the pure clinical testing of the vestibular function may underestimate the rate of OVSs [15]; thus, it is desirable for the future to include instrumental evaluations. Finally, given the nature of the study, no conclusions regarding the role of vestibular dysfunction in migraine pathophysiology can be made. Given the above limitations, these findings may only be regarded as a starting point for designing future ad hoc and well-powered studies to deepen our knowledge on the involvement of the oculo-vestibular system in migraine.

## Conclusion

Vestibular dysfunction has been suggested in migraine, but it still represents a poorly elucidated aspect. To our knowledge, this is the first study reporting the incidence of OVSs in a human migraine model, but the exploratory nature of the study does not allow to draw conclusions on the possible implication of a vestibular dysfunction in migraine pathophysiology. Thus, the main objective of this brief communication is to provide data to facilitate the design of well-powered and prospective studies. Ideally, to further characterize this phenomenon, future studies should: i) include a larger population made of healthy controls, patients with migraine without VM, and patients with VM; ii) adopt a solid double-blind, placebo-controlled design; and iii) provide a thorough clinical and instrumental assessment of the oculo-vestibular function.
